# Draft Genome Sequences of Pseudomonas fluorescens Strains PA4C2 and PA3G8 and Pseudomonas putida PA14H7, Three Biocontrol Bacteria against Dickeya Phytopathogens

**DOI:** 10.1128/genomeA.01503-14

**Published:** 2015-01-29

**Authors:** Jérémy Cigna, Yannick Raoul des Essarts, Samuel Mondy, Valérie Hélias, Amélie Beury-Cirou, Denis Faure

**Affiliations:** a CNRS, Institut des Sciences du Végétal, UPR2355, Saclay Plant Sciences, Gif-sur-Yvette, France; b Comité Nord Plant de Pomme de Terre (CNPPT), Semences, Innovation, Protection Recherche et Environnement (SIPRE), Achicourt, France; c Fédération Nationale des Producteurs de Plants de Pomme de Terre (FN3PT), Recherche, Développement et Promotion du Plant de Pomme de Terre (RD3PT), Achicourt, France; d UMT Innoplant (FN3PT-INRA IGEPP1349), Le Rheu, France

## Abstract

Pseudomonas fluorescens strains PA4C2 and PA3G8 and Pseudomonas putida strain PA14H7 were isolated from potato rhizosphere and show an ability to inhibit the growth of Dickeya phytopathogens. Here, we report their draft genome sequences, which provide a basis for understanding the molecular mechanisms involved in antibiosis against Dickeya.

## GENOME ANNOUNCEMENT

Pectinolytic enterobacteria of the Pectobacterium and Dickeya genera are causative agents of the blackleg and soft rot diseases on potato crops (1). Dickeya populations have mostly been described in tropical environments but were recently considered an emerging pathogen in Europe (2). Until now, Dickeya dianthicola and the new species Dickeya solani (3) are the two Dickeya species associated with blackleg on potato plants in Europe (4). Currently, there is no treatment against blackleg disease; hence, biocontrol strategies are developed in order to reduce symptoms in crops (5, 6). Many Pseudomonas strains are plant growth-promoting rhizobacteria (7) and may produce antibiotic compounds, such as phenazine (8) and lipopeptides (9, 10), which inhibit the growth of other microorganisms. Consequently, Pseudomonas spp. are often used as biocontrol agents of soilborne pathogens worldwide (11). Here, we report the genome sequences of three Pseudomonas strains, Pseudomonas fluorescens PA4C2, P. fluorescens PA3G8, and Pseudomonas putida PA14H7, which were isolated from potato environments and are able to inhibit the growth of Dickeya potato pathogens.

The genome sequence of each strain was established using the Illumina HiSeq 2000 v3 technology. Following a whole-genome shotgun, an 8-kb mate-pair library was designed and used to generate paired-end sequencing reads of 2 × 100 bp. The sequence reads were trimmed based on their quality scores, and ambiguous nucleotides were eliminated. The reads were assembled using CLC Genomics Workbench 5.5 (length fraction, 0.5; similarity fraction, 0.8), and contigs >2,000 bp were collected. Scaffolding was performed using SSPACE basic V2.0 (12), and GapFiller 1.1 was used in order to close the gaps caused by repeat regions (13). Finally, the last step was performed, which consisted of mapping the sequenced reads with each contig end. The mapped reads were assembled and blasted on the contig ends in order to fill the last gaps. The annotation of each genome was accomplished with the Rapid Annotations using Subsystems Technology (RAST) server (14). The genomic features of each Pseudomonas strain are presented in Table 1. The genome sequences of these three antagonistic bacterial strains against Dickeya phytopathogens provide a good basis for further biomolecular analyses in order to study the mechanisms involved in antibiosis.

**Table 1:** Genome features of P. fluorescens strains PA3G8 and PA4C2 and P. putida PA14H7

Strain	Genome size (bp)	Accession no.	*N*_50_ (bp)	No. of contigs	No. of scaffolds	G+C content (%)	No. of CDSs^*a*^	No. of tRNAs	No. of rRNAs
P. fluorescens PA3G8	6,391,599	JBOO00000000	233,515	53	9	58.9	5690	67	16
P. fluorescens PA4C2	6,210,847	AXDA00000000	120,136	88	5	60.2	5442	61	15
P. putida PA14H7	5,878,755	JBOP00000000	173,633	64	7	62.4	5318	63	7

a CDSs, coding DNA sequences.

### Nucleotide sequence accession numbers.

These whole-genome shotgun projects have been deposited in DDBJ/ENA/GenBank under the accession numbers AXDA00000000 (P. fluorescens PA4C2), JBOO00000000 (P. fluorescens PA3G8), and JBOP00000000 (P. putida PA14H7).
